# Protein Profiling and Histone Deacetylation Activities in Somaclonal Variants of Oil Palm (*Elaeis guineensis* Jacq.)

**DOI:** 10.1155/2013/613635

**Published:** 2013-06-03

**Authors:** Jamilah Syafawati Yaacob, Hwei-San Loh, Rosna Mat Taha

**Affiliations:** ^1^Institute of Biological Sciences, Faculty of Science, University of Malaya, 50603 Kuala Lumpur, Malaysia; ^2^School of Biosciences, The University of Nottingham Malaysia Campus, Jalan Broga, 43500 Semenyih, Selangor, Malaysia

## Abstract

Mantled fruits as a result of somaclonal variation are often observed from the oil palm plantlets regenerated via tissue culture. The mantling of fruits with finger-like and thick outer coating phenotypes significantly reduces the seed size and oil content, posing a threat to oil palm planters, and may jeopardize the economic growth of countries that depend particularly on oil palm plantation. The molecular aspects of the occurrence of somaclonal variations are yet to be known, possibly due to gene repression such as DNA methylation, histone methylation and histone deacetylation. Histone deacetylases (HDACs), involved in eukaryotic gene regulation by catalyzing the acetyl groups are removal from lysine residues on histone, hence transcriptionally repress gene expression. This paper described the total protein polymorphism profiles of somaclonal variants of oil palm and the effects of histone deacetylation on this phenomenon. Parallel to the different phenotypes, the protein polymorphism profiles of the mantled samples (leaves, fruits, and florets) and the phenotypically normal samples were proven to be different. Higher HDAC activity was found in mantled leaf samples than in the phenotypically normal leaf samples, leading to a preliminary conclusion that histone deacetylation suppressed gene expression and contributed to the development of somaclonal variants.

## 1. Introduction

**Figure 1:**
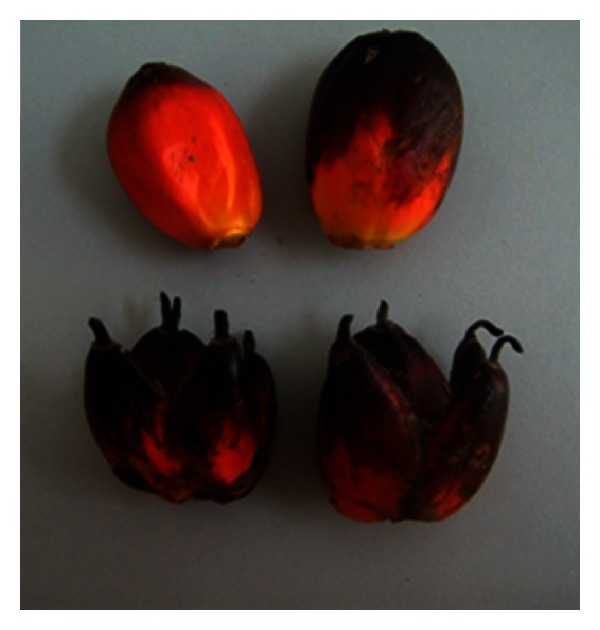
Comparison between phenotypically normal (top) and mantled fruits (bottom). Source: from Advance Agricultural Resources Pty Ltd (AAR).

Mantled fruits in oil palm (*Elaeis guineensis* Jacq.) are a result of somaclonal variation that is often observed when the oil palm plantlets are regenerated via tissue culture [1, 2]. The mantled phenotypes have finger-like fruits and a thick outer coating, hence reducing the seed size and also oil production significantly. The overall size of mantled fruits is generally smaller than the normal, in some cases without seed. The comparison between a phenotypically normal fruit and a mantled oil palm fruit is shown in Figure 1.

The fruit mantling phenomenon has also made the scaling-up process of oil palm clones to be difficult as about 5% of the clonal populations derived from tissue culture exhibits somaclonal variation phenomenon [3]. Those undesirable abnormal phenotypic differences include the development of abnormal flowers where the male parts of the flowers are “feminized” [4]. Specifically, in the case of abortive mantling phenomenon, no pollen is produced by the male inflorescences, and as for female inflorescences, a ring of supplementary carpels is produced surrounding the gynoecium, which in turn prevents the mantled oil palm fruits from ripening [5]. This mantling phenomenon poses a threat to oil palm planters and can further jeopardize the economic growth of countries that depend particularly on oil palm plantation. Therefore, the underlying factors that cause the formation of these somaclonal variants need to be investigated, so that a detection marker can be developed to serve as an early detection method for the mantled fruits. The current study aims to evaluate the involvement of histone deacetylase (HDAC) in the mantling phenomenon and hence brings us one step closer to producing an excellent detection marker at early vegetative stage of the seedling in the future.

Even though somaclonal variation is often reported as a result of tissue culture propagation, the occurrence of somaclonal variation may not be unique to in vitropropagation as it can happen naturally in somatic and reproductive tissues in plants [6], possibly triggered by genomic shock or plasticity. This happens when the plants have exhausted its usual physiological responses to environmental stress [7]. This therefore also explains why somaclonal variation is often produced in tissue culture, where the plants are unable to withstand tissue culture stress. However, there are also other external factors involved in inducing the production of these somaclonal variants, such as the departure from organized meristematic growth, the genetic makeup (genotype, ploidy) of the explant source, the use of plant growth regulators (type and concentration), and also the source of explants [8]. For example, in oil palm propagation via tissue culture, somaclonal variation may arise when flower tissues are used as the explant source [8].

The molecular aspects of the occurrence of somaclonal variation have not yet been fully investigated [1], but one of the most likely factors is gene repression. There are several factors that can result in gene repression such as DNA methylation, histone methylation, and histone deacetylation. Histone deacetylases (HDACs) involve in eukaryotic gene regulation by catalyzing the acetyl groups removal from the lysine residues on histone; hence, HDAC transcriptionally repress gene expression [9–13]. In histone acetylation, the *ε*-amino groups of lysines in the N-terminal domain of core histones are acetylated by histone acetyltransferases (HATs) with acetyl-CoA as the cosubstrate [14]; this type of modification is reverted back by the reaction of histone deacetylases (HDACs). Hence, it can be deduced that histone acetylation results in gene expression, whereas histone deacetylation yields the opposite outcome. 

HDACs play the opposite role of HATs, whereby it is related to transcriptional repression and involved in gene silencing [15]. In plants, there are three families of HDAC, namely, the *RPD3/HAD* gene family, the *HD2* enzymes family (maize histone deacetylases) and the sirtuin family that is associated with yeast *SIR2* [15, 16]. The *SIR2* proteins are eukaryotic *NAD+* dependent protein deacetylases that are involved in many important biological processes such as DNA repair, transcriptional modulation, and life span control [17]. Plants also have another HDAC type called the *HD2*-type deacetylases that is only unique to plants and is unrelated to the other three HDAC types [15]. HDAC often work together with DNA methyltransferases and *HMT*s in their action [15]. Examples on the effects of HDAC reaction include the experimental study of overexpression of rice HDAC1 that resulted in a boost to growth rate and a striking phenotypic change in rice [15]. In experiments conducted on *Arabidopsis*, mutations of the genes that encode for *Rpd3*-type HDAC *HDA6* showed that they were involved in gene silencing, while antisense inhibition of *HD2*-type HDAC leads to seed abortion [15]. There are also other examples on HDAC activity that have been observed in other plants, but all of them also imply that HDAC repress gene transcription and hence also repress gene expression [15].

Tian et al. (2005) suggested that histone acetylation and deacetylation reactions were actually reversible, promoter-dependent, and also locus specific, hence enabling an excellent control over gene regulation in response to developmental changes and environmental stimuli [11]. Therefore, due to the reversible nature of histone deacetylation process, this implies that the mantling phenomenon can be reversed over time, as shown by several oil palm trees [18]. However, the occurrence of somaclonal variation in oil palm would still cause a great loss, hence it is very important that the mantling phenomenon be detected at an earlier stage by using a detection marker. The present study aims to demonstrate the relationship of HDAC enzyme levels and protein profiles involved in the mantling phenomenon.

## 2. Materials and Methods

### 2.1. Sample Collection

Two categories of samples were used in this study, namely, the phenotypically normal fruits and the somaclonal variants (mantled fruits), where different parts of the trees were sampled: the leaves, fruits, and florets. All samples were collected from AAR (Applied Agricultural Resources Pty Ltd) oil palm plantation in Paloh Substation, Johor, Malaysia, with the help of AAR researchers (Advanced Agriecological Research). Six sample categories were studied including 100% abortive clonal mantled palm (AM), 50% fertile clonal mantled palm (FM), androgynous clonal palms (AD1 and AD2),and normal clonal palms (N1 and N2 and with 4 or more stigmas).

**Figure 2:**
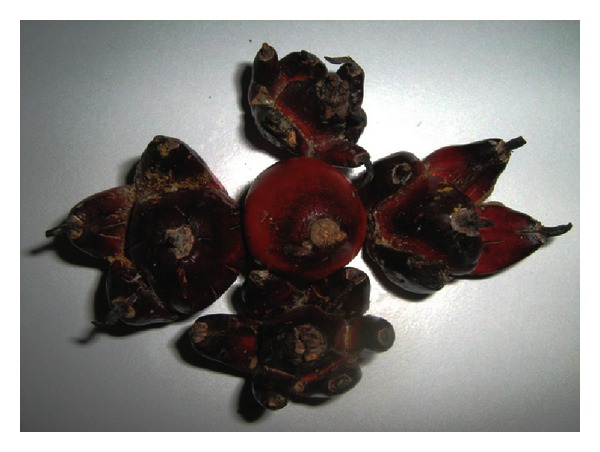
Different degree of mantling (number of “finger”). Source: from AAR.

The mantling phenomenon can be visually observed at different levels, in terms of the number of “finger” present and the degrees of mantling (either 100% abortive mantled or 50% fertile mantled). Overall, the 100% abortive mantled fruits are generally smaller than the 50% fertile mantled fruits. This is because the 100% abortive mantled fruits would be aborted before they become mature, and therefore the collected fruits were smaller. In this study, only the “five-fingers” fruits were used in the protein extractions. The mantled fruits also have a different number of “finger,” compared to one another although they might come from the same tree. Some of them may have four, five, or even six “finger”, as shown in Figure 2.

### 2.2. DNA Extraction and SSR Analysis

Frozen leaves (2 g) of clonal lines of oil palm trees were ground to powder form using a mortar and liquid nitrogen. Modified CTAB method was employed in DNA extraction experiments, whereby PVP-40, ascorbic acid, DIECA, and 2-mercaptoethanol were added to the extraction solvent. The extracted DNA was subjected to SSR analysis by using 9 degenerate primers [19] to prove their clonal origin.

### 2.3. Total and Nuclear Protein Extraction

 60 mg of frozen leaf, fruit (mantled and normal), and floret samples was ground to a fine powder using a mortar and liquid nitrogen. Total protein extraction from the tissue samples was done using “Plant Total Protein Extraction Kit” (Sigma-Aldrich) and subjected to protein concentration assays (Pierce 660 nm Protein Assay (Thermo Scientific)), followed by subsequent SDS-PAGE analysis to allow for the visualization of their protein profiles. Nuclear protein extraction was also carried out from leaf tissues (20 g) using “Plant Nuclei Isolation/Extraction Kit (CelLytic PN)” (Sigma-Aldrich).

### 2.4. HDAC Analysis and ELISA Assay

 The extracted nuclear protein extracts (900 mg) were subjected to HDAC activity assay using EpiQuik HDAC Activity/Inhibition Assay Kit (Epigentek). In this part of the research, the histone deacetylase enzyme activity was measured by means of enzyme-linked immunosorbent assay (ELISA) at 450 nm. The HDAC level of all sample categories were compared.

### 2.5. Statistical Analysis

Assessment of results was conducted using randomized complete block design (RCBD) with 3 replicates and statistically analyzed using analysis of variance (ANOVA), whereby mean comparisons were done using Duncan's multiple range test (DMRT) with the least significant differences at 5% level.

## 3. Results and Discussion

### 3.1. DNA Extraction and SSR Analysis

**Figure 3:**
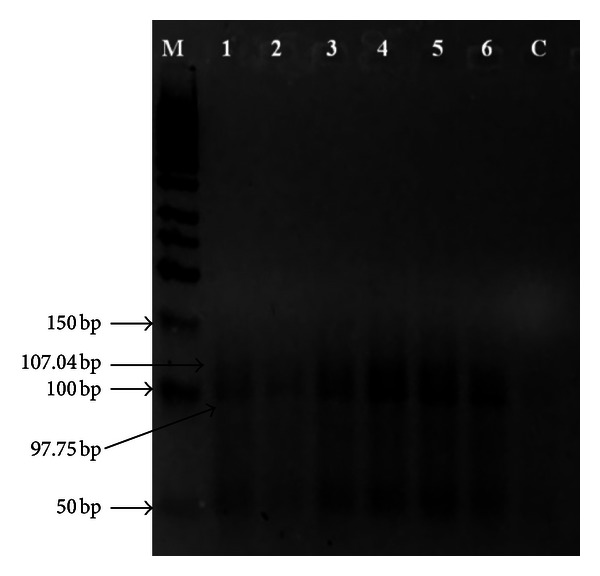
Amplification profile of SSR primer P1T6 (1: AM, 2: FM, 3: AD1, 4: AD2, 5: N1, 6: N2, M: Fermentas GeneRuler 50 bp DNA ladder, and C: control).

**Figure 4:**
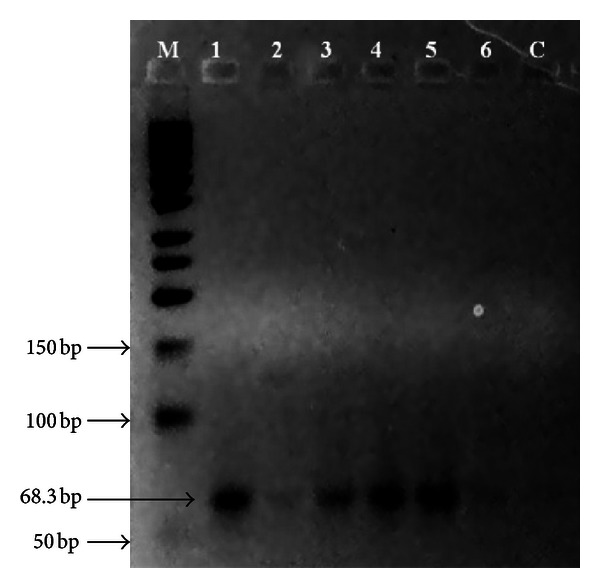
Amplification profile of SSR primer P4T10 (1: AM, 2: FM, 3: AD1, 4: AD2, 5: N1, 6: N2, M: Fermentas GeneRuler 50 bp DNA ladder, and C: control).

Two out of nine primers (primer P1T6 and P4T10) gave good results verifying that all samples were clonal siblings, as shown in Figures 3 and 4.

### 3.2. Total Protein Profiling and HDAC Analysis

**Figure 5:**
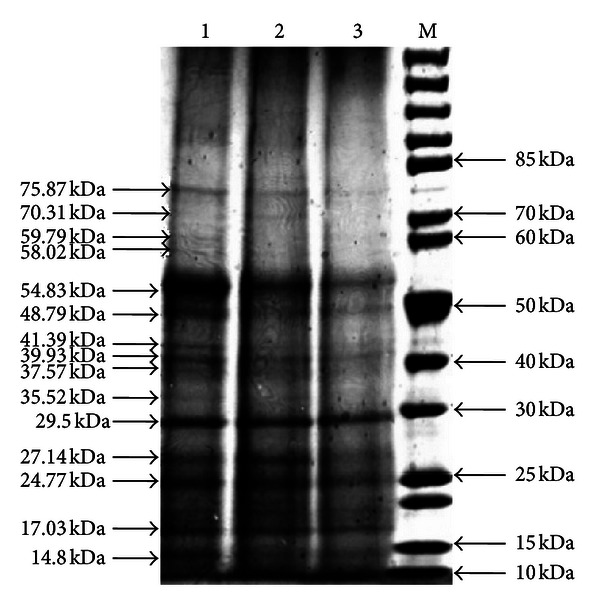
Total protein profiles of leave (1: phenotypically normal, 2: 50% mantled, 3: 100% mantled, and M: PageRuler Unstained Protein Ladder).

**Figure 6:**
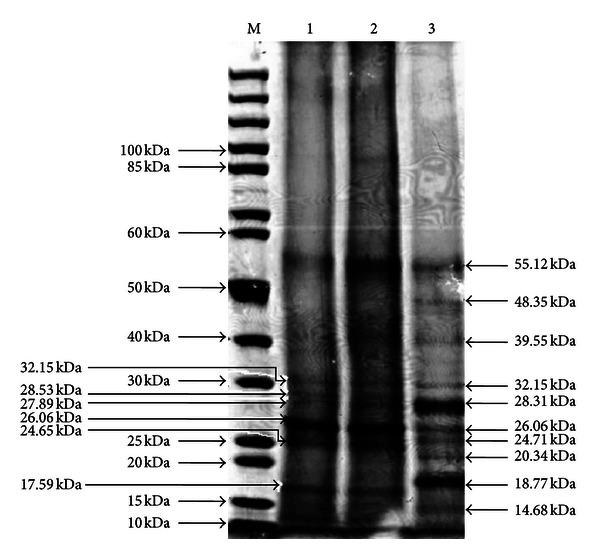
Total protein profile of fruits (1: 100% mantled, 2: 50% mantled, 3: phenotypically normal, and M: PageRuler Unstained Protein Ladder).

**Figure 7:**
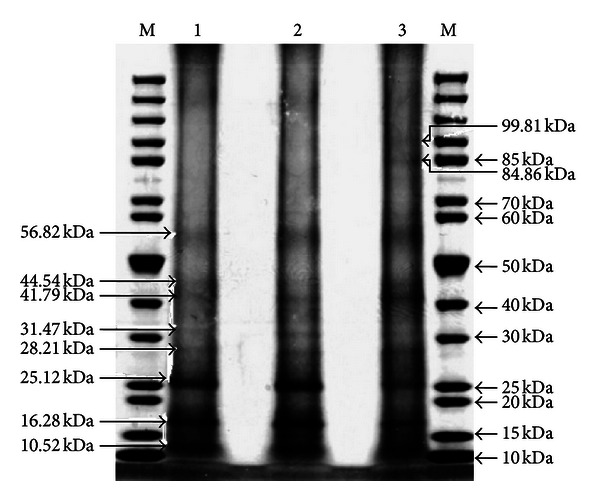
Total protein profile of florets (1: 100% mantled, 2: 50% mantled, 3: Phenotypically normal, and M: PageRuler Unstained Protein Ladder).

Figures 5, 6, and 7 show the total protein profiles of the leaves, fruits, and florets, respectively as electrophoresed in SDS-PAGE gels. The electrophoresed protein fragments from the leaves were similar in all three samples (100% abortive mantled, 50% fertile mantled, and phenotypically normal). However, some of the electrophoresed protein fragments from the fruit and floret samples of 100% abortive mantled and 50% fertile mantled were different from those of the phenotypically normal.

As observed in Figure 6, there was one prominent band that was present in all three fruit samples, which was about 55.12 kDa in size. Other than that, the phenotypically normal fruit also had two other unique prominent bands present, which were about 28.31 kDa and 18.77 kDa in size, while both the 100% and 50% abortive mantled fruits also had another one prominent band, which was about 26.06 kDa in size. This 26.06 kDa protein band was also present in the total protein profile of the phenotypically normal fruit, but that band was much fainter than that of the 100% and 50% abortive mantled fruits. 

More interestingly, both the 100% and 50% abortive mantled fruits had four protein bands specifically unique for both of the samples, and that particular protein bands were not present in the phenotypically normal fruit protein profile. Those four bands were about 28.53 kDa, 27.89 kDa, 24.65 kDa, and 17.59 kDa in size. These findings might be due to the upregulation on certain amino acid synthesis, which should not usually occur, like in the phenotypically normal fruit [20, 21]. Besides that, there were also seven protein bands that were unique in the protein profile of the phenotypically normal fruit but were completely absent from the protein profile of the mantled fruits. Those seven bands were about 48.35 kDa, 39.55 kDa, 28.31 kDa, 24.71 kDa, 20.34 kDa, 18.77 kDa, and 14.68 kDa in size. 

As for the florets, the banding patterns for all the samples were similar, except for two particular bands that were present in the phenotypically normal floret sample but absent from both the 100% mantled and 50% mantled florets (Figure 7). Those two bands were about 99.81 kDa and 84.86 kDa in size. The banding patterns of the protein polymorphism profiles of all three categories were summarized in Table 1.

**Figure 8:**
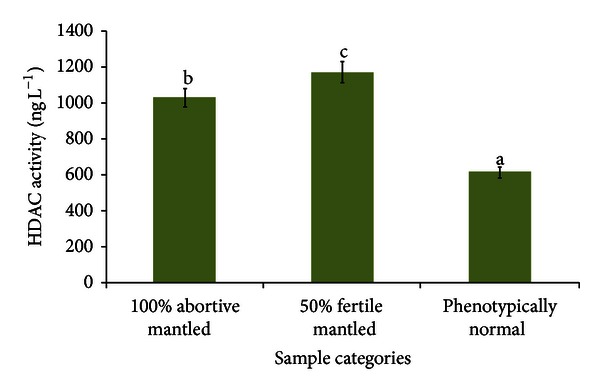
Average HDAC activity levels of leaf samples. Mean values with different letters are significantly different at *P* < 0.05.

The average HDAC activity levels of both 100% and 50% mantled samples were significantly higher than the HDAC activity level of the phenotypically normal sample, where 1030.869 ng/mL and 1173.888 ng/mL of average HDAC activity levels were recorded for 100% and 50% mantled samples respectively, while 614.557 ng/mL of average HDAC activity level was recorded for the phenotypically normal sample (Figure 8). 

The mantling phenomenon undergoes an epigenetic regulation with similar underlying genomic sequences in all kinds of plant tissues; changes are produced at the gene expression level. This study aimed to investigate the involvement of HDAC enzyme in fruit mantling phenomenon, whereby the target was the chromatin (DNA and histones) inside the nucleus. The chromatin content of the cells would be similar despite the different tissues of leaves, florets and fruits. It is of greater interest and preference to determine the somaclonal variations at earlier time especially during the vegetative stage, rather than the reproductive stage after 4-5 years of growth, which latter has wasted time, manpower, and money. With the general aim of development of an early detection biomarker and the availability of more straightforward nuclear protein extraction method from the leaves, the HDAC activity assay was conducted on the leaf samples. 

Out of the total 9 primers [19] used in the SSR analyses, two primers (primer P1T6 and P4T10) gave good results, whereas some of the other primers did not even produce any amplification, possibly due to the fact that those primers were degenerate primers [19]; hence, although they did work on the oil palm leaf samples in the previous study, they might not work on oil palm with slightly different genomes. As observed from Figures 3 and 4, all of the samples produced bands of similar size (~50 bp and ~100 bp in size for Primer P1T6 and about 63.5 bp in size for Primer P4T10), but with different degree of band intensities. Hence, it can be deduced that all of the six trees sampled in the study were of the same clonal origin and genotype.

The different total protein polymorphism profiles attributing to mantling morphologies with different severity (50% versus 100% mantled) were successfully shown for the leaves, fruits, and florets of the 100% mantled, 50% mantled, and phenotypically normal trees. As observed in Table 1, all three leaf samples produced similar banding patterns (Figure 5) although some of the bands appeared fainter than the comparable bands from the other samples. However, in contrast to the leaf total protein profile, the banding patterns of both the 100% mantled and 50% mantled fruits and florets (Figures 6 and 7) were similar, but both of them had different banding patterns as compared to the phenotypically normal fruits and florets, as clearly depicted in Table 1. This might be due to housekeeping genes inside the fruits and florets that may have been upregulating and downregulating the synthesis of certain proteins [20, 21], and hence causing the proteins that should have been present in a phenotypically normal fruits and florets to be absent in mantled fruit and floret samples instead. This may also cause the synthesis of new proteins in the mantled tree that were not present in a phenotypically normal tree. 

More importantly, the alteration of the proteins was targeted in the fruits but not in the leaves. Although those seven and those two protein bands were present in the phenotypically normal fruits and florets (Table 1), respectively, their absence in the mantled fruits and florets indicated that the mantling phenomenon occurs in the absence of certain proteins resulting from gene repression during protein synthesis which correlated with the findings by Tian et al. (2005) in their study on *Arabidopsis thaliana* [11]. These protein polymorphism profiles indicated that the mantling phenomenon was tissue specific and occurrence of somaclonal variations had started as early as at the floret developmental stage. This could lead to the speculation that the variations might even be detected during floral initiation and floral organ developmental stages. However, further investigations will be conducted in order to prove this. The differences of the protein profiles were very valuable for further investigation on the specific proteins involved in the mantling phenomenon, from which the identity of the different protein fragments present could be established and hence serve as a guideline to investigate the developmental stage and protein synthesis pathway involved in the mantling phenomenon.

Based on the findings of this study, it was suggested that histone deacetylation was causing this phenomenon, where the enzyme histone deacetylase (HDAC) had been involved in eukaryotic gene regulation by catalyzing the acetyl groups removal from the lysine residues on histone, and hence transcriptionally repressed gene expression [22]. This therefore disturbed the normal transcription and translation processes involved in the different developmental pathways which should have occurred (like in the phenotypically normal fruits), therefore causing certain amino acids (which would be normally produced) not to be produced, while some other amino acids which should be absent in the phenotypically normal fruits to be synthesized. 

This hypothesis was supported by the different HDAC activity shown by the nuclear proteins of mantled and phenotypically normal leaves. As shown in Figure 8, theHDAC activity levels of both the 100% abortive mantled and 50% fertile mantled were significantly higher than that of the phenotypically normal leaves. Hence, it could be deduced that the HDAC enzyme had caused a certain extent of gene repression in the mantled samples because it works oppositely with the HAT, which had contributed to the acetylation and switching on the gene(s) to be expressed. However, the HDAC activity level of the 50% fertile mantled was slightly higher than that of the 100% abortive mantled. This might be due to the inclusion of the nonhistone proteins in the nucleus for the function of HDAC [23]. Therefore, as we studied the HDAC enzyme activity as a whole but not the mantling expression-specific one, there were some non-histone protein activities concurrently taking place and had contributed to the elevated HDAC level. This might have induced a higher level of HDAC activity in the 50% fertile mantled than the 100% abortive mantled. Besides, a different site of lysine deacetylation might contribute to the gene repression effects. Nevertheless, histone deacetylation is jeopardizing the normal protein synthesis that should have occurred, thus causing certain proteins not to be synthesized [15]. The disruption of the normal development process should have caused the mantling abnormalities to occur, whereby it could be speculated that the absence of the seven (fruits) and two (florets) protein bands from the mantled fruits and florets might be the result of histone deacetylation, which had caused the expression of these proteins' coding genes to be switched off.

Generally, this study had facilitated the establishment of protein polymorphisms between oil palm somaclonal variants, which could serve as a principle protocol for further investigations in proteomics. More experiments such as different protein extraction/fractionation methods and two-dimensional SDS-PAGE will be conducted on more oil palm samples in the future in order to further verify the protein polymorphisms as mentioned in this paper. Besides, the protein bands of interest could be excised from the gel and digested for mass spectrometric analysis for their characteristics and identities.

## 4. Conclusions

In this study, the different protein polymorphism profiles of the somaclonal variants involved in the mantling phenomenon have been clearly elucidated. Based on the HDAC activity assay, it was shown that histone deacetylation did involve in the fruit mantling phenomenon. 

## Figures and Tables

**Table 1:** Estimated molecular size of protein fragments of 100% abortive mantled, 50% fertile mantled, and phenotypically normal samples (leaf, fruit, and floret).

Leaf			Fruit			Floret		
AM (kDa)	FM (kDa)	N (kDa)	AM (kDa)	FM (kDa)	N (kDa)	AM (kDa)	FM (kDa)	N (kDa)
								99.81
								84.86
75.87	75.87	75.87						
70.31	70.31	70.31						
59.79	59.79	59.79						
58.02	58.02	58.02						
						56.82	56.82	56.82
			55.12	55.12	55.12			
54.83	54.83	54.83						
48.79	48.79	48.79						
					48.35			
						44.54	44.54	44.54
						41.79	41.79	41.79
41.39	41.39	41.39						
39.93	39.93	39.93						
					39.55			
37.57	37.57	37.57						
35.52	35.52	35.52						
			32.15	32.15	32.15			
						31.47	31.47	31.47
29.50	29.50	29.50						
			28.53	28.53				
					28.31			
						28.21	28.21	28.21
			27.89	27.89				
27.14	27.14	27.14						
			26.06	26.06	26.06			
						25.12	25.12	25.12
24.77	24.77	24.77						
					24.71			
			24.65	24.65				
					20.34			
					18.77			
			17.59	17.59				
17.03	17.03	17.03						
						16.28	16.28	16.28
14.80	14.80	14.80						
					14.68			
						10.52	10.52	10.52

AM: 100% abortive mantled, FM: 50% fertile mantled, and N: phenotypically normal.
